# Differential Transcriptional Responses of Human Granulocytes to Fungal Infection with *Candida albicans* and *Aspergillus fumigatus*

**DOI:** 10.3390/jof9101014

**Published:** 2023-10-14

**Authors:** Tilman E. Klassert, Martin Hölzer, Cristina Zubiria-Barrera, Julia Bethge, Esther Klaile, Mario M. Müller, Manja Marz, Hortense Slevogt

**Affiliations:** 1 Respiratory Infection Dynamics, Helmholtz Centre for Infection Research—HZI Braunschweig, 38124 Braunschweig, Germany; cristina.zubiriabarrera@helmholtz-hzi.de (C.Z.-B.); slevogt.hortense@mh-hannover.de (H.S.); 2 Department of Respiratory Medicine and Infectious Diseases, Hannover Medical School, German Center for Lung Research (DZL), BREATH, 30625 Hannover, Germany; 3 Methodology and Research Infrastructure, Genome Competence Center (MF1), Robert Koch Institute, 13353 Berlin, Germany; hoelzerm@rki.de; 4 ZIK Septomics, Host Septomics, Jena University Hospital, 07747 Jena, Germanyesther.klaile@med.uni-jena.de (E.K.); mario.mueller1@med.uni-jena.de (M.M.M.); 5 RNA Bioinformatics and High Throughput Analysis, Friedrich Schiller University Jena, 07743 Jena, Germany; manja@uni-jena.de

**Keywords:** neutrophils, *Candida*, *Aspergillus*, RNAseq

## Abstract

Neutrophils are critical phagocytic cells in innate immunity, playing a significant role in defending against invasive fungal pathogens. This study aimed to explore the transcriptional activation of human neutrophils in response to different fungal pathogens, including *Candida albicans* and *Aspergillus fumigatus*, compared to the bacterial pathogen *Escherichia coli*. We identified distinct transcriptional profiles and stress-related pathways in neutrophils during fungal infections, highlighting their functional diversity and adaptability. The transcriptional response was largely redundant across all pathogens in immune-relevant categories and cytokine pathway activation. However, differences in the magnitude of differentially expressed genes (DEGs) were observed, with *A. fumigatus* inducing a lower transcriptional effect compared to *C. albicans* and *E. coli*. Notably, specific gene signatures associated with cell death were differentially regulated by fungal pathogens, potentially increasing neutrophil susceptibility to autophagy, pyroptosis, and neutrophil extracellular trap (NET) formation. These findings provide valuable insights into the complex immunological responses of neutrophils during fungal infections, offering new avenues for diagnostic and therapeutic strategies, particularly in the management of invasive fungal diseases.

## 1. Introduction

Neutrophils, the most abundant leukocytes in human blood [1], are professional phagocytic cells that play a crucial role in innate immunity, particularly in the defense against invasive fungi [2]. Clinical evidence has long supported their prominent contribution in controlling the early stages of fungal infection through an array of antifungal effector mechanisms. These include the production of reactive oxygen intermediates, release of antimicrobial enzymes, and formation of neutrophil extracellular traps (NETs) [3,4]. Notably, patients with primary (e.g., chronic granulomatous disease) or acquired (e.g., undergoing immunosuppressive therapy) neutrophil deficiency are highly susceptible to fungal infections [2,5], underscoring the significance of neutrophils in antifungal immunity. Among the fungal pathogens encountered by neutrophils, *Candida albicans* and *Aspergillus fumigatus* stand out as the most common species infecting humans. These fungi can cause a wide range of infections, from superficial to life-threatening systemic infections, especially in immunocompromised individuals [2].

For a long time, and until recently, neutrophils were considered as poorly plastic and transcriptionally inactive cells. Recent research has challenged the conventional view of neutrophils as short-lived effectors with limited adaptability, unveiling the functional diversity and flexibility of these immune cells [6,7]. This includes the transcriptional activation of these cells when challenged with *C. albicans* yeast [8,9] and hyphae [9], resulting in altered expression of genes encoding transcriptional regulators, receptors, and cytokines. In contrast, the transcriptional response against *A. fumigatus* remains largely unknown in neutrophils.

Although initial data on the transcriptional activation of neutrophils against fungi have been reported, there remains a significant need to understand the comprehensive transcriptional programming of these central immune cells. In this study, we explored the transcriptional activation of human neutrophils against fungi in a comparative approach. Granulocytes were challenged with the two fungal pathogens *C. albicans* and *A. fumigatus* and their transcriptional activation compared to the response elicited by the bacterial pathogen *Escherichia coli*. A better understanding of the functional diversity of neutrophils during fungal infections may contribute to the development of innovative diagnostic and improved therapeutic strategies.

## 2. Materials and Methods

Study design: This study was designed to explore the ability of neutrophils to respond at transcriptional level to different fungal pathogens (*C. albicans* and *A. fumigatus*) as compared to their response to a bacterial strain (*E.coli*), which was used as reference pathogen for immune activation. An overview of the workflow is shown in the Supplementary Materials (Supplementary Figure S1). Neutrophils were isolated from fresh blood of four healthy donors and subjected to in vitro stimulation with the fungal and bacterial pathogens. RNAseq data allowed then for a comprehensive characterization of the transcriptional response.

Neutrophils isolation: Human polymorphonuclear (PMN) cells were isolated from 500 mL of fresh whole blood (drawn within 1 h before use) of four healthy male donors. Blood was layered onto an equal volume of the gradient material 1-Step Polymorphs (Accurate Chemical & Scientific Corporation, New York, NY, USA). After centrifugation at 650× *g* for 35 min at RT, distinct leukocyte bands and a cell pellet became visible. The second fraction, comprising PMNs, was cautiously retrieved and mixed in a 1:1 ratio with a solution of 0.45% NaCl. Subsequently, this mixture was subjected to centrifugation at 400× *g* for a period of 10 min at 4 °C, followed by hypotonic lysis to eliminate any contaminating erythrocytes. The neutrophils were then pelleted again, using centrifugation at 400× *g* for 10 min at 4 °C, and subsequently washed once with sterile phosphate-buffered saline (PBS). Cell viability of >95% was assessed with trypan blue staining. Cell fraction purity of >98% was assessed with specific surface markers using flow cytometry.

Preparation of fungi and bacteria: For *C. albicans* (SC5314), overnight culture in a YPD medium was followed by triple washing with PBS. The yeast cells were then resuspended at a concentration of 10^8^ yeasts/mL in RPMI 1640 GlutaMAX medium supplemented with 10% fetal bovine serum (FBS; Biochrom, Berlin, Germany). To obtain *A. fumigatus* (AF293) conidiospores, the fungus was cultured on AMM plates at 30 °C for 6 days. After harvesting the conidiospores by rinsing the plates with water containing 0.05% Tween-20 (Sigma-Aldrich, Hamburg, Germany), a single-cell suspension was obtained via filtering through 70 μm and 30 μm pre-separation filters (Miltenyi Biotec, Woking, UK). The conidia were then washed twice in PBS and resuspended at a concentration of 10^7^ conidia/mL in RPMI 1640 GlutaMAX medium supplemented with 10% FBS (Biochrom, Berlin, Germany). To generate germlings, the conidia were incubated at 37 °C under continuous shaking for 6–8 h. Subsequently, they were centrifuged and resuspended at 108 cells/mL in fresh RPMI 1640 GlutaMAX medium supplemented with 10% FBS. For *E. coli* (isolate 018:K1:H7) cultures, an overnight LB medium growth was washed three times in PBS before being resuspended in RPMI 1640 GlutaMAX medium supplemented with 10% FBS. The bacterial concentration was adjusted to 10^9^ cfu/mL. All pathogens were rendered non-viable via heat-killing through incubation at 65 °C for 30 min, after which they were immediately used for the stimulation assays. Pathogens were heat-killed to obtain an immune activation based on the specific interaction of pathogen-associated-molecular-patterns (PAMPs) and patter-recognition-receptors (PRRs). Heat-killing might not only increase the exposure of certain PAMPs [10] but may also reduce the variability between the in vitro assays that result from different metabolic activities and growth rates between pathogens.

Stimulation assays: PMNs from the four donors were resuspended at 10^7^ cells/mL in RPMI 1640 GlutaMAX medium (Gibco, Grand Island, NY, USA) supplemented with 10% FBS (Biochrom, Berlin, Germany) and 1% Penicillin/Streptomycin (Thermo Fisher Scientific, Waltham, MA, USA). They were seeded on 6-well plates (VWR International, Darmstadt, Germany) and allowed to equilibrate at 37 °C and 5% CO_2_ for 1 h. Cells were then stimulated with heat-killed pathogens at pathogen/host ratios of 1:1 for *C. albicans* yeast and *A. fumigatus* germ tubes and 10:1 for *E. coli*. After 3 h of incubation at 37 °C and 5% CO2, PMN cell viability > 90% was assessed with trypan blue staining, and the neutrophils were subjected to RNA isolation.

RNA sequencing: RNA was isolated from 10^7^ PMNs using the RNeasy Mini Kit (Qiagen, Hilden, Germany). A subsequent step was implemented to eliminate any residual genomic DNA by employing DNaseI (Qiagen, Hilden, Germany). The quantification of total RNA was carried out using a Nanodrop ND-1000 spectrophotometer (Thermo Fisher Scientific, Waltham, MA, USA). To assess the RNA samples’ quality, the RNA Integrity Number (RIN) values were determined (>7) using a Tape Station 2200 (Agilent Technologies, Santa Clara, CA, USA). Poly-(A) RNA purification was performed from 2 μg of total RNA utilizing the Dynabeads mRNA DIRECT Micro Purification Kit (Thermo Fisher Scientific, Waltham, MA, USA), following the manufacturer’s instructions. High Sensitivity RNA Screen Tapes (Agilent Technologies, Santa Clara, CA, USA) were used for quality control to verify the successful depletion of rRNA. To create strand-specific whole transcriptome libraries, the Ion Total RNA-Seq Kit v2.0 (Thermo Fisher Scientific, Waltham, MA, USA) was employed. The purified RNA was fragmented using RNAse III, and Ion adapters were ligated to the resulting fragments. Reverse transcription was conducted using the SuperScript III Enzyme Mix (Thermo Fisher Scientific, Waltham, MA, USA). Barcoded primers were utilized to amplify the libraries, employing the Platinum PCR High Fidelity polymerase (Thermo Fisher Scientific, Waltham, MA, USA). The final barcoded libraries’ size distribution analysis and quantification were performed using D1000 Screen Tapes on the Tape Station 2200 (Agilent Technologies, Santa Clara, CA, USA). Ion Sphere particles and the Ion PI Hi-Q Chef Kit were then used for library template amplification on the Ion Chef instrument (Thermo Fisher Scientific, Waltham, MA, USA). These library templates were then loaded onto Ion PI Chips and sequenced on an Ion Proton Sequencer (Thermo Fisher Scientific, Waltham, MA, USA). A total of 16 samples were multiplexed on 4 Ion chips for the sequencing process, yielding 10.1–27.7 million reads with a mean length of 99 bp. The raw sequence data in fastq format have been deposited in the Sequence Read Archive (SRA) at the National Center for Biotechnology Information (NCBI) and can be accessed via the NCBI homepage (https://www.ncbi.nlm.nih.gov/, accessed on 13 October 2023; accession number: PRJNA1003649).

Bioinformatic analysis of RNA-seq data: Quality check and trimming of raw sequence data. The initial raw data in fastq format underwent quality assessment using FastQC v0.11.3 [11] to ensure data integrity. Subsequently, the reads were trimmed with a window size of 10 using Prinseq [12]. After removing reads corresponding to rRNA with SortMeRNA, the trimmed reads were aligned to the human genome version GRCh38 using Segemehl v0.2.0 [13], following the approach described in Riege et al. [14] with default parameters and the addition of the --splits option. To quantify the mapped reads at the exon level with strand specificity, HTSeq-Count (v0.6.0) [15] was employed. The full human Ensembl annotation (version GRCh38.80), encompassing both protein-coding and non-coding genes, was utilized as the reference.

Analysis of differential gene expression and key pathway identification: The mapped reads were counted per gene and per sample at the exon level and used as input for statistical analyses carried out with DESeq2 v1.10.1 [16] and various Bioconductor [17] packages in R. To filter out low-expressed mRNAs and account for transcript length biases in normalized read counts, transcripts per kilobase million (TPM) were calculated. Subsequently, for each stimulatory setting, genes with TPM > 5 were kept for further expression analyses as described elsewhere [18,19]. The contrasts explored in this study always included the unstimulated samples as controls. Thus, pairwise comparisons were conducted to investigate the impact of each pathogen stimulation by comparing unstimulated samples with pathogen-stimulated samples. Differentially expressed genes (DEGs) that exhibited significant regulation (Padj < 0.05) and an absolute log2 fold change (FC) > 1 were subjected to GO-enrichment analysis using g:Profiler [20]. Additionally, pathway analyses of selected GO-categories were performed with the Ingenuity Pathway Analysis (IPA) tool from Qiagen (Hilden, Germany), using default settings (significance threshold: −log (*p*-value) cutoff of 1.3). The RNAseq results obtained for PMNs in this study were further analyzed in comparison to the transcriptional data retrieved from similar stimulation assays with monocytes, as described elsewhere [21].

## 3. Results

**Figure 1:**
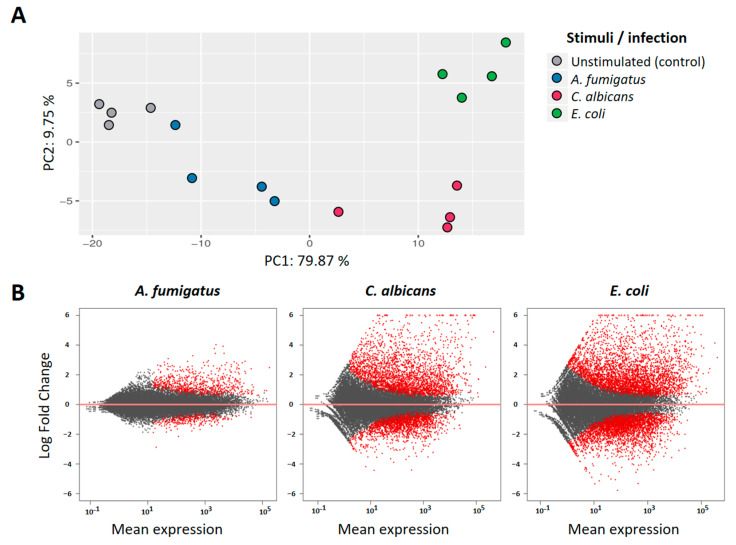
Transcriptome profile in neutrophils upon challenge with three different pathogens. (**A**) Principal component analysis (PCA) of the top 100 most variant genes. The first two principal components (PC1–PC2) account for over 98% of the variance in the dataset. (**B**) Scatter plots depicting the mean expression and log2 fold changes of differentially expressed genes (DEGs) in response to each of the pathogens. Red dots represent significantly regulated genes.

Analysis of the RNAseq data revealed a high number of genes that were significantly regulated in PMNs after co-culture with the different pathogens. Principal component analysis (PCA) of the 100 most informative genes showed a distinct pattern for each of the stimuli when compared to the unstimulated controls (Figure 1A). The first component (PC1), accounting for almost 80% of the observed variance, retrieved a similar spatial-pattern for *C. albicans* and *E.coli* stimulations, while *A. fumigatus*-stimulated samples were found closer to the controls. Analysis of fold change distributions of DEGs (Figure 1B) confirmed this observation, as the magnitude of the transcriptional regulation after *A. fumigatus* stimulation was significantly lower when compared to the other two pathogens.

This relative low transcriptional activation by *A. fumigatus* was identified as a cell-specific trait, as the same experimental setting retrieved no significant variance in the transcriptional activation profiles when stimulating monocytes from the same donors (Supplementary Figure S2, detailed data on monocyte assays in Klassert et al. [21]). To investigate to which extent the immune responses against the pathogens were cell-specific, comparative analyses were made on the regulation of immune relevant genes (Gene ontology category GO:0002376—Immune System Process) between monocytes and neutrophils. Significant differences in the regulatory profiles could be observed (Supplementary Figure S3), suggesting an important number of cell-specific orchestration programs of the immune response in each of the cell types.

**Figure 2:**
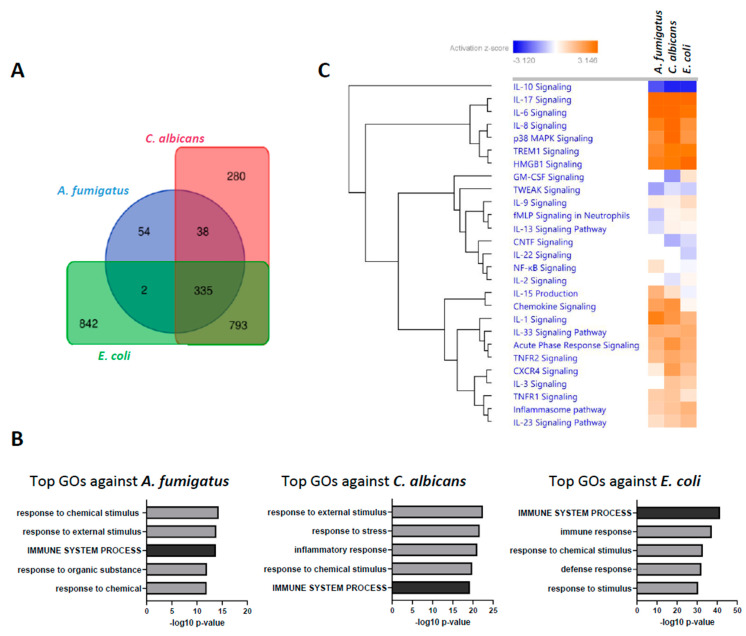
Overlap of different pathogen-induced transcriptional profiles in human PMNs. (**A**) Venn diagram showing the intersections of significant DEGs with the different pathogens: *A. fumigatus* (blue), *C. albicans* (red), and *E. coli* (green). (**B**) GO enrichment analyses for the genes regulated by each pathogen. Shown are the top 5 regulated biological processes. (**C**) Analysis of the activation of cytokine signaling pathways (IPA). Shown are the significantly enriched pathways (−log (*p*-value) cutoff of 1.3) hierarchically clustered by their activation Z-scores achieved upon co-incubation with each pathogen.

To further characterize the transcriptional responsiveness of PMNs, we compared the differential expression profiles induced by each of the pathogens. *E. coli* stimulation accounted for a total of 1972 DEGs (Padj < 0.05; abs(log2FC) > 1) in our dataset, followed by *C. albicans* with 1446 regulated genes. In comparison, *A. fumigatus* only induced the regulation of 429 DEGs with these same thresholds. Comparative analysis of the DEGs shows, however, a huge overlap of this regulated gene-set, with 335 DEG shared among the three stimulation settings (Figure 2A). Moreover, Gene ontology (GO) enrichment analysis retrieved similar categories for all pathogens, with the Immune System Process (GO:0002376) among the top five biological processes in all stimulations (Figure 1B). Furthermore, comparable Log2FC profiles could be observed in in genes of this GO category in pairwise comparisons between pathogens (Supplementary Figure S4). Activation analysis of the most relevant cytokine pathways further confirmed this observation (Figure 2C), suggesting a high degree of redundancy in the transcriptional regulation of the immune response against all three pathogens.

**Figure 3:**
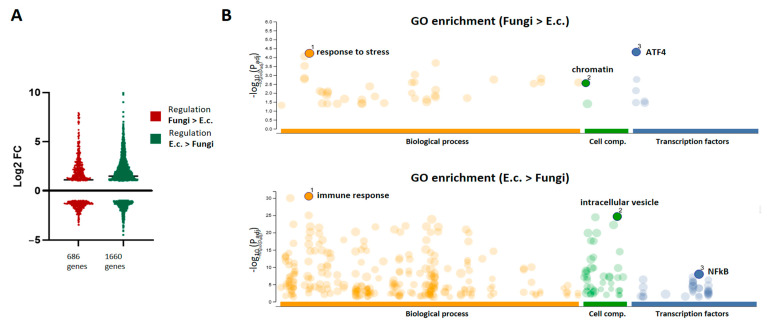
DEGs with differential regulation magnitudes upon fungal or bacterial PMN challenge. (**A**) Shown are the Log2-FC profiles of genes with stronger regulation upon fungal challenge (686 genes) or upon bacterial challenge with *E. coli* (1660 genes). (**B**) GO enrichment analyses for the gene clusters with stronger fungal or bacterial (*E. coli*) responsiveness. Shown are the significantly enriched biological processes, cellular components, and transcription factors in each gene cluster, highlighting the top GO categories in each case.

Although the regulation of immune programs was highly redundant between pathogens, we aimed to explore the differential transcriptional responses activated by the fungal pathogens in comparison to the bacterial agent. Thus, sets of genes were identified with higher FC regulation after fungal stimulation (686 genes) or after bacterial activation (1660 genes) when compared with each other. The expression profiles retrieved similar patterns of up- and down-regulated genes in both sets (Figure 3A). Interestingly, GO enrichment analysis showed highly dissimilar regulation profiles between both sets (Figure 3B). The gene-set with stronger regulation upon bacterial stimuli retrieved GO categories related to the immune response, with the intracellular vesicles as central cellular component and the subunits of NFkB as main transcriptional factors linked to the regulation of these genes. In contrast, “response to stress” was the most enriched GO category of the genes preferentially regulated by the fungal pathogens, with binding sequences for the Activating Transcription Factor (ATF) family as the most involved regulatory elements.

**Figure 4:**
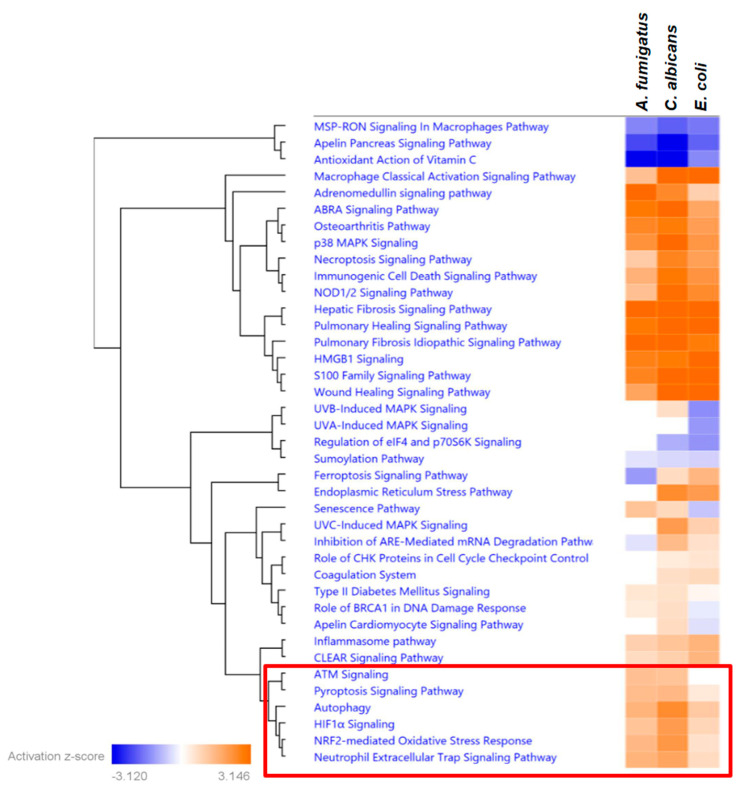
Differential analysis of the activation of stress-related pathways (response to stress). Shown are the significantly enriched pathways (−log (*p*-value) cutoff of 1.3) hierarchically clustered by their activation Z-scores achieved upon co-incubation with each pathogen. The red box displays the clusters of pathways with increased activation scores upon fungal challenge when compared to stimulation with *E. coli*.

Analysis of the pathways related to the response to stress revealed a high level of shared activation patterns between all three pathogens. However, we also identified a set of pathways with increased activation Z-scores upon fungal stimulation when compared to the stimulation with *E. coli* (Figure 4). These pathways included several cell death mechanisms, including Autophagy, Pyroptosis, and the Neutrophil Extracellular Trap formation, also known as NETosis. A deeper analysis of the top genes, leading to the differential activation profile between bacterial and fungal pathogens, allowed the identification of central regulator genes in each cell death pathway, such as *CREB5* and *LRRK1* in the autophagy, the pyroptosis-relevant inhibitor of apoptosis protein (NAIP) or the Selectin P-encoding gene *SELP*, which is important in the NETosis (see Supplementary Figure S5).

## 4. Discussion

This study provides further evidence for the relevance of transcriptional programs in the orchestration of complex immunological responses by human neutrophils. To the best of our knowledge, this is the first study in this cell type addressing comparative transcriptional activation assays against different pathogens. Our results depict a transcriptional response against fungal and bacterial agents that are largely redundant in the immune programs induced by each pathogen but highly variable in the magnitude of the transcriptional activation by the different pathogens.

While *C. albicans* and *E. coli* induced a comparable transcriptional response in PMNs as measured by the amount and fold change range of the regulated genes (1446 and 1972 genes, respectively), the *Aspergillus* germ tubes induced a significant lower transcriptional effect (429 genes). Such differences were not observed when monocytes of the same donors were subjected to identical stimulatory assays [21], suggesting potential cell-specific differences between monocytes and PMNs in their efficiency to sense *Aspergillus* germ tubes. This might be dependent on different PRRs repertoires on these cell types or a dissimilar ability to effectively transduce the signal. Since transcriptional regulation is a result of pathogen recognition using specialized PRRs, the stimulation assays in this study were designed to provide a similar pathogenic surface as input in each stimulatory setting. Thus, equal multiplicity of infection (MOI = 1) was employed for the fungal pathogens (comparable size), while an MOI = 10 was used for the bacterial stimulation (smaller size). However, PAMPs might have an unequal distribution on the surface of the pathogens. In fungi, these PAMPs include ß-glucans, mannose, and α-mannose [22], which are then recognized by PRRs of the neutrophils such as Dectin-1 [23], Dectin-2 [24], and Mincle [25]. It is plausible that differential distributions of both the PAMPs on the fungi or the PRR on the immune cells may lead to the dissimilar transcriptional responsiveness observed between both fungal stimulations in neutrophils (but not monocytes). Moreover, differential roles have already been described for the same PRRs (e.g., Mincle) in the two cell subsets, leading to distinct antifungal responses when comparing monocytes and neutrophils [26]. In addition, neutrophils have shown a higher dependency than monocytes on complement activation for the inflammatory response to fungi [27], which might also contribute to the cell-specific differences observed in the non-opsonic conditions of our experiments.

Despite the variance observed in the magnitude of DEGs between the different pathogen stimulations, the GO-enrichment analysis retrieved a high overlap of the biological processes engaged by all three pathogens. This relative redundancy was especially notorious in immune-relevant categories, as the analysis of cytokine pathway activation also retrieved comparable results between all stimulatory settings. Nevertheless, the expression analyses in different pathogenic settings revealed distinct variations in the transcriptional profiles, such as evident differences in the PCAs. These distinctions could arise from the pathogens being recognized differentially by specific PRRs, indicating that innate immunity may not be entirely non-specific, aligning with insights from other cell types [28].

When searching for specific transcriptional patterns that define neutrophil responses against fungal pathogens, we discovered a significant cluster of stress-related pathways with distinct features related to cell death. This finding enabled the identification of specific gene signatures associated with cell death, which appear to be uniquely regulated using fungal pathogens and might translate into an increased susceptibility of neutrophils toward autophagy, pyroptosis, and NETosis. Interestingly, genes that were differentially regulated upon fungal or bacterial stimulation included the only gene (*LRRK1*) with an antagonistic expression signature between fungal and bacterial stimulations (up-regulated by *C. albicans* and down-regulated by *E. coli*). This gene has been described as an important regulator of autophagy [29]. Recent studies have subscribed the protective effect of autophagy in fungal infections [30], which in other myeloid cells has been attributed to an enhanced fungicidal activity, including the expression of ROS and the increased efficiency of phagocytosis [31]. The observed regulation toward pyroptosis is in line with studies showing *Candida*-mediated activation of this pathway in macrophages [32,33] and might be part of a fungal-driven immune escape mechanism [33]. Interestingly, upon fungal stimulation, we also detected significant activation scores for the NET-osis pathway, a neutrophil-specific defense mechanism by which extracellular NETs composed of DNA–histone complexes and proteins are actively released to trap pathogens [34]. The relevance of de novo gene expression programs for NETosis is controversially discussed in the literature. While Sollberger et al. [35] described it as transcription-independent process, Kahn and Palaniyar reported a suppression of NETosis when blocking transcription [36]. Thus, it is unclear whether certain transcriptional programs might contribute to NETosis, and the importance of DNA transcription in dying neutrophils might still remain an enigma. However, in cancer research, two recent studies were able to identify novel expression signatures of NET-related genes with prognostic value [37,38], suggesting a certain transcriptional control at least in non-inflammatory diseases. Interestingly, three of these NET-signature genes (*CREB5*, *SELP,* and *SELPLG*) were also differentially regulated between bacterial and fungal stimuli in our dataset. Our results depict a differential regulation of several NET-related genes. It still needs to be clarified to which extent the transcriptional signatures might contribute to the actual event of NETosis. However, the increased activation of this pathway using fungi seems biologically plausible, especially since neutrophils are able to sense microbe size and have been shown to selectively release NETs in response to large pathogens [39].

This work has some limitations, which include the use of heat-killed pathogens. Thus, the study only addresses differences in pathogen-specific PAMP-PRR interactions but does not cover the impact of metabolic and active immune escaping mechanisms by these pathogens. Further investigations using live pathogens should confirm the findings raised in this study.

In conclusion, our study reveals fungal-induced transcriptional signatures that may lead to a differential regulation of cell death in granulocytes. A better understanding of the mechanisms that drive neutrophil cell death during infection might have important clinical relevance, especially for invasive fungal disease management, since dysregulation of neutrophil cell death has been recently associated with sepsis [40].

## Data Availability

Raw sequence data in fastq format have been deposited in the Sequence Read Archive (SRA) at the National Center for Biotechnology Information (NCBI) and can be accessed via the NCBI homepage (https://www.ncbi.nlm.nih.gov/, accessed on 13 October 2023; accession number: PRJNA1003649).

## Supplementary Materials

The following supporting information can be downloaded at: https://www.mdpi.com/article/10.3390/jof9101014/s1, Figure S1: Experimental workflow, Figure S2: Comparison of transcriptional regulation profiles, Figure S3: Comparative analysis of immune-relevant expression profiles between neutrophils and monocytes, Figure S4: Comparative analysis of immune-relevant expression profiles induced in PMNs by the different pathogens, Figure S5: Differential analysis of cell death pathways activation.
