# Supplementary material for: Differential Transcriptional Responses of Human Granulocytes to Fungal Infection with Candida albicans and Aspergillus fumigatus
Source: J Fungi (Basel). 2023 Oct 14;9(10):1014. doi: 10.3390/jof9101014 (PMC10607568; doi:10.3390/jof9101014)
Supplement: Supplementary file 1 [file jof-09-01014-s001.zip › jof-2607626-supplementary.pdf]

SUPPLEMENTAL MATERIAL

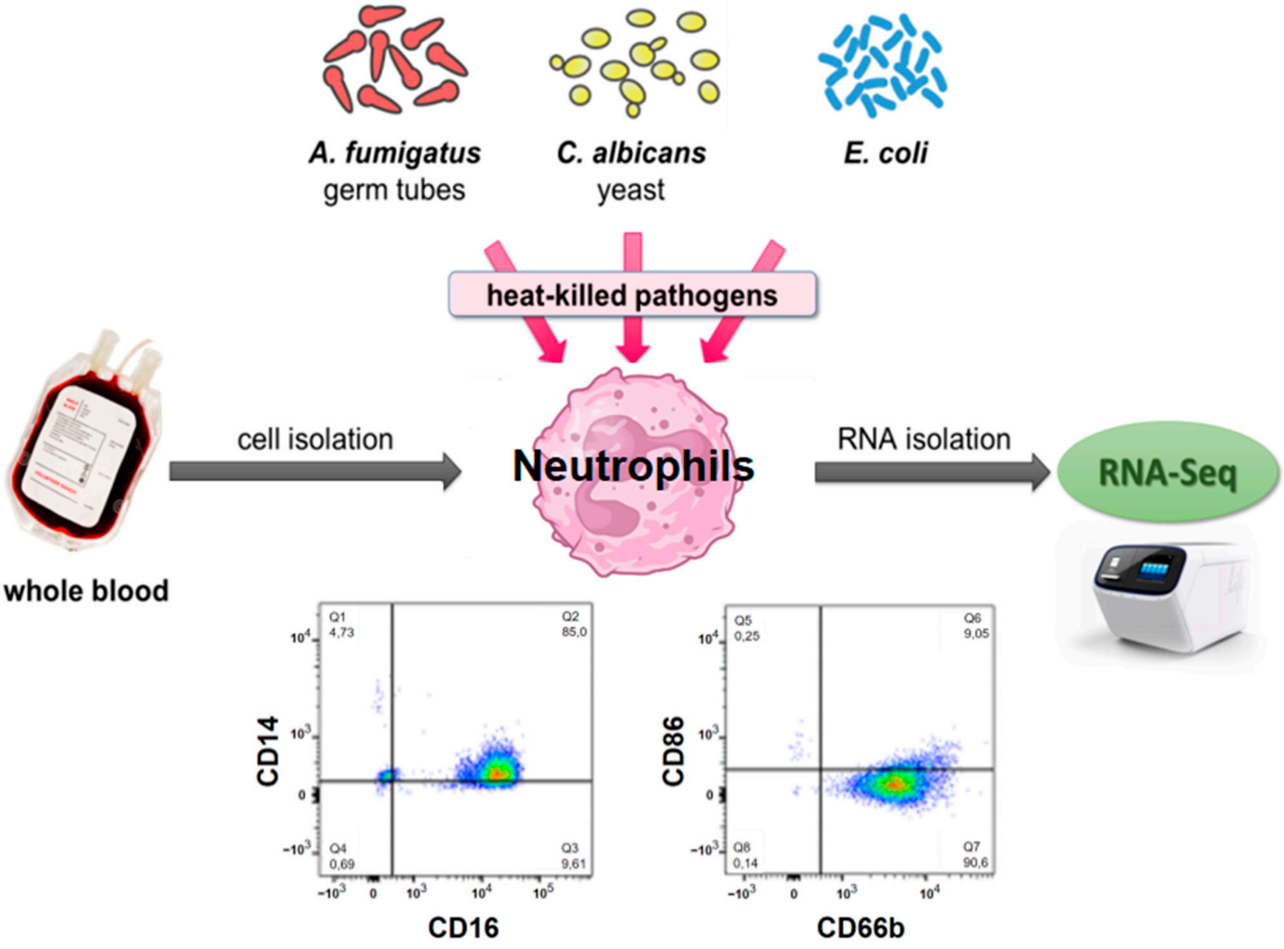

Suppl. Figure S1.- Experimental workflow. Human neutrophils were isolated from fresh whole blood and purity of the cells was validated by flow cytometry. Neutrophils were then challenged for 3 h with heat-killed pathogens (*A. fumigatus, C. albicans* or *E. coli).* RNA was isolated from the neutrophils and subjected to RNA sequencing and expression profiling analyses.

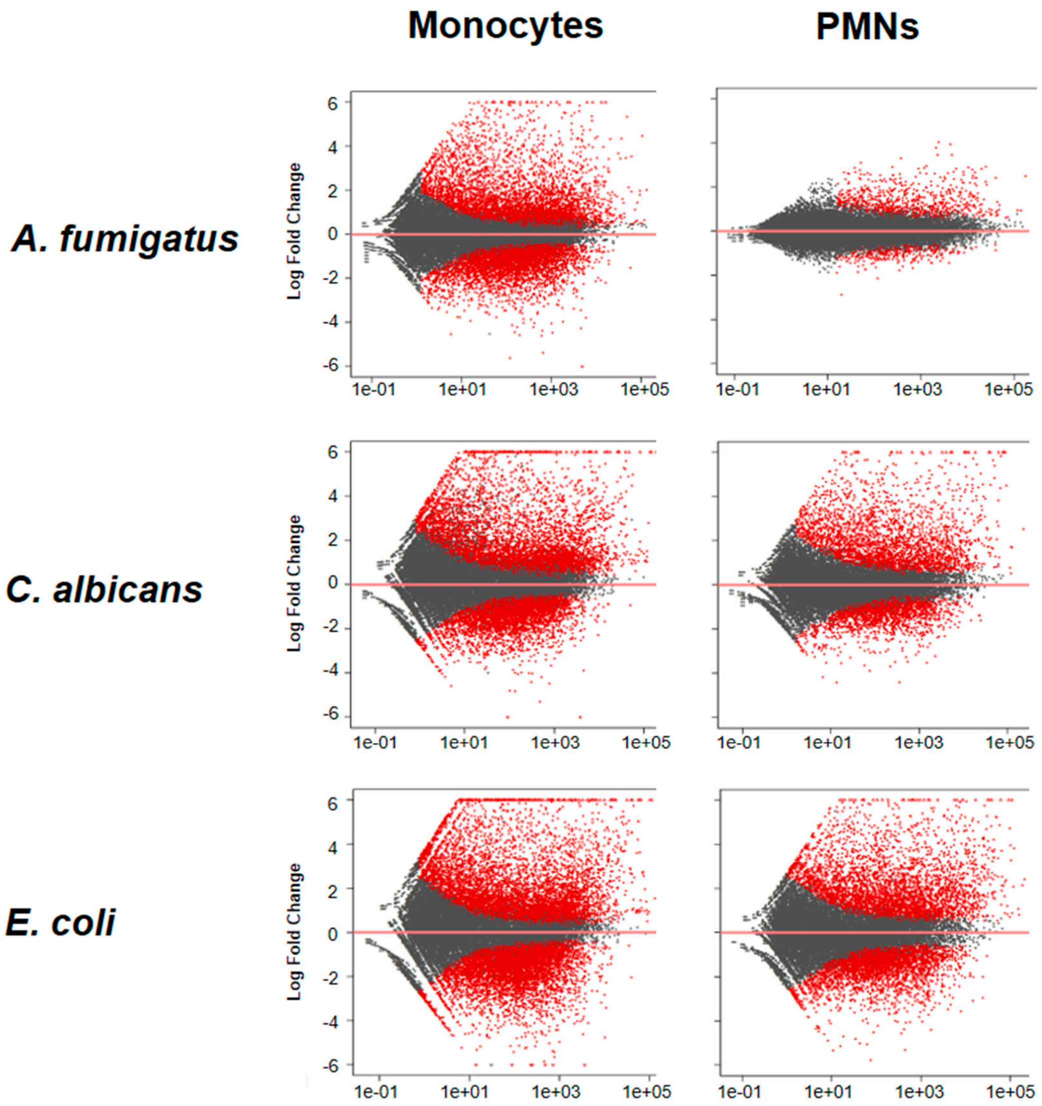

Suppl. Figure S2.- Comparison of transcriptional regulation profiles achieved by different pathogens in human monocytes and neutrophils. Scatterplots depicting the mean expression and log2 fold changes of differentially expressed genes (DEGs) in response to each of the pathogens for both cell types. Red dots represent significantly regulated genes.

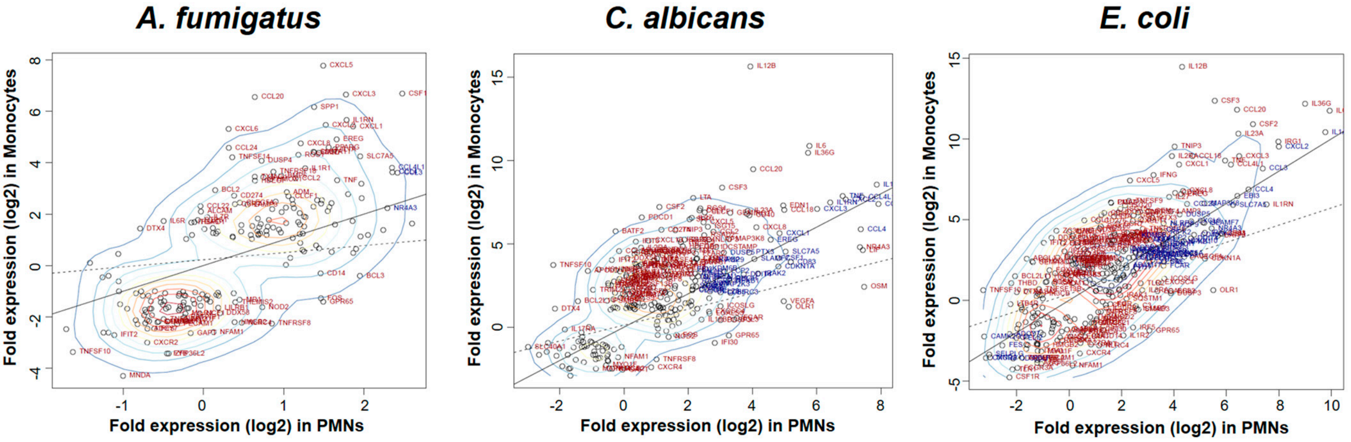

Suppl. Figure S3.- Comparative analysis of immune-relevant expression profiles between neutrophils and monocytes. Shown are scatter plots with the differential transcriptional effects of each pathogen on PMNs (horizontal axis) and monocytes (vertical axis). The genes depicted belong to the GO category GO:0002376 (Immune System Process). The different immune cells were isolated from the same donors and stimulated in parallel for 3 hours. Comparative analyses depict the coordinates of each gene based on the fold changes reached in each cell model as compared to unstimulated controls. Genes written in red indicate significant differences in the magnitude of their regulation between cell types.

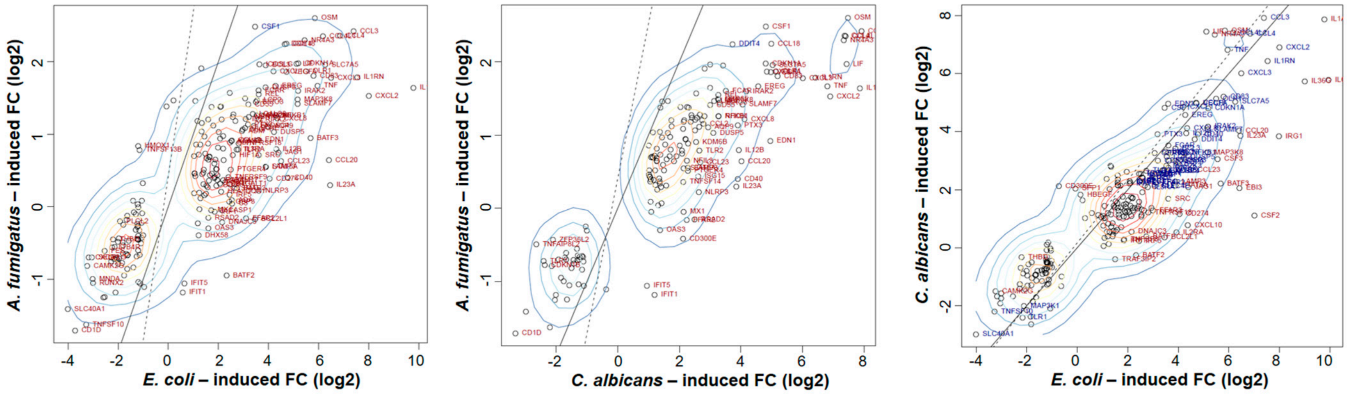

Suppl. Figure S4.- Comparative analysis of immune-relevant expression profiles induced in PMNs by the different pathogens. Shown are scatter plots with pairwise comparisons of the transcriptional changes induced by different pathogens on PMNs. Depicted are all DEG belonging to the GO category GO:0002376 (Immune System Process). Each gene coordinates was plotted based on the fold changes reached after the specific stimulation with each of the pathogens. Genes written in red indicate significant differences in the magnitude of their regulation between infections.

A

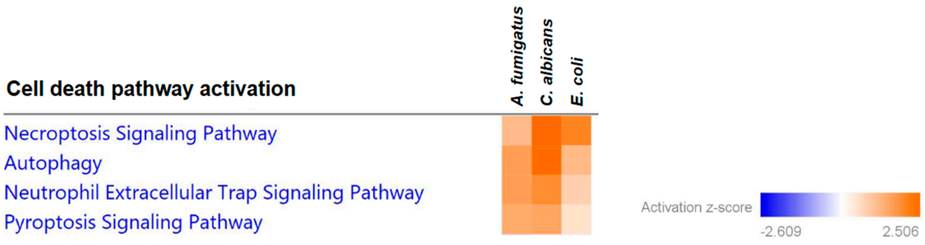

B

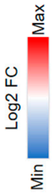

Necroptosis

	A.f.	*C.a.*	*E.c.*
TNFSF10	-1,629	-2,207	-3,274
FADD	ns	-1,965	-1,944
CASP8	ns	-1,618	-1,975
PYCARD	-1,04	-1,273	-1,196
TAB1	ns	-1,382	-1,236
RIPK3	ns	ns	-1,614
TRADD	ns	-1,058	ns
TP53	ns	ns	1,272
TNFRSF10A	ns	ns	1,298
PPID	ns	ns	1,344
IKBKB	ns	ns	1,95
TAB2	ns	ns	2,079
TNFRSF1B	ns	1,283	1,938
CASP1	ns	1,088	2,139
CYLD	ns	1,416	1,912
PLA2G4C	ns	1,514	2,642
CFLAR	ns	1,874	2,411
SLC25A13	ns	1,066	3,254
BIRC2	ns	1,859	2,56
TNIP1	ns	1,756	3,335
NLRP3	ns	2,635	3,611
BIRC3	1,441	3,311	3,493
PPIF	2,01	3,865	2,93
TICAM1	1,924	3,552	4,834
TNF	1,666	6,811	5,955

Autophagy

	A.f.	*C.a.*	*E.c.*
CREB5	-1,011	-1,788	-3,272
PIK3C2B	-1,069	-1,629	-1,433
WIPI1	ns	-1,485	-2,293
WIPI2	ns	-1,9	-1,767
TFEB	ns	-1,394	-1,454
HGF	ns	ns	-2,315
MAPK1	ns	ns	-1,883
TGFA	ns	ns	-1,628
ATG2B	ns	ns	-1,485
DAPK2	ns	ns	-1,472
ATM	ns	ns	-1,343
AKT1S1	ns	ns	-1,311
ULK1	ns	ns	-1,271
LRRK1	ns	1,027	-1,003
ATG7	ns	ns	2,659
ARL8B	ns	1,695	2,325
DDIT3	1,137	2,06	1,475
TBK1	ns	1,873	2,976
RIPK2	1,198	2,742	3,613
NFKB2	1,728	2,926	3,803
NFKB1	1,255	3,278	4,292
TICAM1	1,924	3,552	4,834
VEGFA	1,858	4,973	4,718
SLC7A5	1,952	5,033	6,256
TNF	1,666	6,811	5,955

Pyroptosis

	A.f.	*C.a.*	*E.c.*
TLR6	ns	-2,411	-3,092
NLRP12	ns	-1,694	-2,921
TLR1	ns	-2,41	-2,162
NAIP	ns	-1,314	-2,47
NLRP6	ns	-1,81	-1,802
CASP8	ns	-1,618	-1,975
PYCARD	-1,04	-1,273	-1,196
TLR10	ns	ns	-2,19
TLR9	ns	ns	-1,947
GSDMB	ns	ns	-1,821
NLRP1	ns	ns	-1,49
PRKACB	ns	ns	-1,081
ELAVL1	ns	ns	-1,019
CASP9	ns	1,049	ns
CASP4	ns	1,165	1,857
CASP1	ns	1,088	2,139
TRAF3	ns	1,404	2,068
GBP1	ns	2,286	2,462
NLRP3	ns	2,635	3,611
IL1R1	1,268	2,391	2,595
NFKB2	1,728	2,926	3,803
NFKB1	1,255	3,278	4,292
TNF	1,666	6,811	5,955
IL1B	1,906	7,745	7,615
IL1A	1,638	7,869	9,78

NETosis

	A.f.	*C.a.*	*E.c.*
SELPLG	ns	-1,924	-3,048
CXCR2	ns	-1,675	-3,174
LCAT	ns	-1,007	-2,561
SELP	ns	ns	-2,803
PLCL2	ns	ns	-1,927
MAPK1	ns	ns	-1,883
RAF1	ns	ns	-1,302
PLCB2	ns	ns	-1,24
PRKCA	ns	ns	-1,204
ITGAL	ns	ns	-1,2
PIK3CB	ns	ns	-1,186
AKT1	ns	ns	-1,175
MTOR	ns	ns	-1,062
MCL1	ns	1,036	ns
ARG2	ns	1,805	ns
NLRP3	ns	2,635	3,611
MAP2K3	1,252	2,817	3,524
NFKB2	1,728	2,926	3,803
CXCL8	ns	4,12	4,351
FCAR	1,598	3,513	3,603
PPIF	2,01	3,865	2,93
NFKB1	1,255	3,278	4,292
COL1A1	1,915	4,182	4,379
TNF	1,666	6,811	5,955
IL1B	1,906	7,745	7,615

Suppl. Figure S5.- Differential analysis of cell death pathways activation. (A) Stress-related cell-death pathways and the activation Z-scores achieved upon co-incubation with each pathogen. **(B)** Top 25-DEGs in each of the cell-death pathways. Shown are the log2 fold changes of significantly up- (red) and down- (blue) regulated genes. (ns = not significant)
